# A Return to the Sextant—Maritime Navigation Using Celestial Bodies and the Horizon

**DOI:** 10.3390/s23104869

**Published:** 2023-05-18

**Authors:** Joshua J. R. Critchley-Marrows, Daniele Mortari

**Affiliations:** 1 School of Aerospace, Mechanical and Mechatronic Engineering, University of Sydney, Sydney, NSW 2006, Australia; joshua.critchley-marrows@sydney.edu.au; 2 Aerospace Engineering, Texas A&M University, College Station, TX 77843-3141, USA

**Keywords:** celestial navigation, alternative PNT, maritime

## Abstract

Satellite navigation over recent decades has become the default and, in some cases, sole source of positioning for maritime vessels. The classic sextant has been all but forgotten by a significant number of ship navigators. However, recent risks to RF-derived positioning by jamming and spoofing have resurfaced the need to train sailors again in the art. Innovations in space optical navigation have long been perfecting the art of using celestial bodies and horizons to determine a space vessel’s attitude and position. This paper explores their application to the much older ship navigation problem. Models are introduced that utilize the stars and horizon to derive latitude and longitude. When assuming good star visibility conditions on the ocean, the accuracy delivered is at the 100 m level. This can meet requirements for ship navigation in coastal and oceanic voyages.

## 1. Introduction

The sextant could be argued to be one of the most influential inventions of the second millennium. Invented by John Bird in 1759, and consisting of an optical telescope and some measuring tools, the user could calculate the angle from the star or Sun in relation to the Earth’s horizon. Alongside knowledge of the time, a geographical position could be derived within a few kilometres, allowing humans to travel, explore, transport, and supply the globe with far greater confidence and efficiency.

In the last decades, this indispensable tool has been rejected for satellite-based navigation systems. The first of these systems was TRANSIT, a constellation of approximately five satellites that transmitted Radio Frequency (RF) communication signals to provide a position reference to US Naval missiles and vessels via measuring the Doppler change.

The system’s success led to a modernisation that became known as Global Positioning System (GPS), a constellation of 32 satellites in Medium Earth Orbit (MEO) that regularly transmit their current location as an open service to users across the globe. Unfortunately, even though it is easily adaptable, they are also easily penetrable through jamming and spoofing attacks by altering the data so that a receiver device appears in a different location.

The maritime sector in particular has been subject to some of the most extreme jamming and spoofing attacks of recent years. One such series of events was exposed in a study by the Centre for Advanced Defence Studies [1]. Focusing on GPS spoofing attacks in Russia and Syria, cases were reported in the Black Sea and Syria, where cargo ships reported their position several miles outside of Moscow. Similar events have also been reported in the Port of Shanghai, China [2], as well as around the Red Sea [3].

In response to these technological attacks, as well as other sources of disruption to GNSSs, the International Authority of Lighthouse Authorities derived a suggested list of minimum maritime user requirements for each phase of voyage [4]. The suggested performances suitable for the use of the new generation of sextants are during the ocean and coastal phases of the voyage, where accuracies of less than 100 m are required. This is within an absolute frame, so dead reckoning-based systems would not be feasible.

Alternatives to navigation in the maritime domain have been treated by various authors [5,6,7,8,9,10]. Solutions consider the use of the new communication signals from Very High Frequency (VHF) Data Exchange and Automatic Identification Systems for ranging as well as radar-based and autonomous sextants.

In contrast to most celestial navigation cases considered in the literature [10,11,12,13], which all use modern interpretations of the sextant first developed in antiquity, this paper treats the problem from a spacecraft navigation perspective. The ocean, in the absence of currents, tides, waves, etc., follows the geoid surface. The geoid altitude’s deviation from the ellipsoidal representation of the Earth is in the range of ±100 m, while the local direction normal to the geoid differs from the normal to the ellipsoid up to several arc-secs. This normal deviation difference is, in general, provided in terms of two angles, the north–south deviation and the east–west deviation. The difference is minimal compared to the ellipsoid’s major and minor axes.

This paper first introduces a mathematical foundation to the horizon problem and then adapts it to various estimation formulations to achieve the best accuracy for the various geometries encountered. The foundation is based on models created for the case of a spacecraft in orbit, but powerful simplifications may be made to improve performance in the maritime domain. The approach is justified by a simple but valid model to portray the oceanic environment, which generally follows the ellipsoid.

The paper might be treated as a sequel to [14], which considers the terrestrial celestial navigation problem using a pair of two orthogonal inclinometers instead of a horizon sensor. Even though the horizon is less available than the Earth’s gravity, it is not affected by the motion of the ship, which may even occur while stationary by the roll of ocean waves.

## 2. Horizon Models and Interpretations

This section provides an introduction to the interpretation of the ocean horizon using the conventions presented in [15,16,17]. These are presented in the context of the marine environment.

### 2.1. Earth’s Shape Description

As is well known, the Earth’s shape is not a sphere but more closely resembles an oblate spheroid. The oblate spheroid is commonly described by the World Geodetic System (WGS). Since its development in 1984 by the National Geospatial-Intelligence Agency, using predecessor models from the initial WGS 60 through to WGS 72, the geodetic description has continued to be refined using the latest results in geodetic positioning measurements, land gravity surveys, altimetry data records, and satellite observations. The model presented and considered in this work is the description as of 2014 [18].

The mathematical description of the ellipsoid is a three-dimensional surface in which all cross-sections create either a circle or an ellipse on the plane. The ellipsoid is characterized by three principal axes, each with a radius dimension given by *a*, *b*, and *c*. Convention treats a>b>c. A point on the surface (X,Y,Z) is expressed by

(1) $$\frac{X^{2}}{a^{2}}+\frac{Y^{2}}{b^{2}}+\frac{Z^{2}}{c^{2}}=1.$$

This expression might also be given in matrix form:

(2) $$\left\{\begin{array}{ccc} X, & Y, & Z \end{array}\right\}\left[\begin{array}{ccc} a^{-2} & 0 & 0\\ 0 & b^{-2} & 0\\ 0 & 0 & c^{-2} \end{array}\right]\left\{\begin{array}{c} X\\ Y\\ Z \end{array}\right\}=p_{p}^{T}\;A_{p}\;p_{p}=1,$$

where $p_{p}={\left\{\begin{array}{ccc} X, & Y, & Z \end{array}\right\}}^{T}$ and subscript *p* indicates it is in the ellipsoid or planetary reference frame.

**Figure 1:**
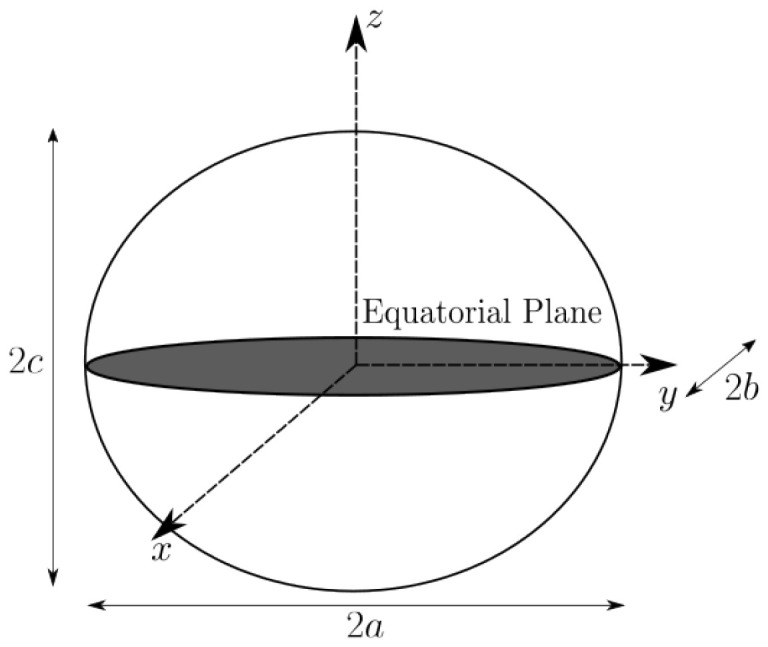
Shape and coordinates of the Earth ellipsoid in the planetary *p* or ECEF frame.

In the case of Earth, the shape is an axial-symmetric ellipsoid, and so the two principal dimensions are a=b. The planetary frame is fixed to the planet and can be made coincident with the Earth Centered, Earth Fixed (ECEF) reference frame. The ellipsoid of the Earth is illustrated in Figure 1.

Taking things a step further, the Earth’s shape is better modelled by the geoid. The geoid is the gravitational potential level that the ocean would conform to in absence of an external force. This may otherwise be labelled as the mean sea level.

The geoid surface is described by the Earth Gravitational Model (EGM), which is a formulation managed by the National Geological Survey in the United States. The EGM represents the gravitational potential due to the Earth’s mass alone (ignoring all rotation effects) by the use of spherical harmonics. The gravitational potential is expressed as

(3) $$V=\frac{\mu }{r}\left[1+\sum_{n=2}^{N}{\left(\frac{a}{r}\right)}^{n}\sum_{m=0}^{n}{\bar{P}}_{nm}\left(\sin\theta \right)\;\left({\bar{C}}_{mn}\cos m\lambda +{\bar{S}}_{mn}\sin m\lambda \right)\right],$$

where μ is the geocentric gravitational constant, *r* is the distance from the Earth’s centre of mass, *a* is the semi-major axis of the WGS-84 ellipsoid, ${\bar{C}}_{nm}$ and ${\bar{S}}_{nm}$ are fully normalized gravitational coefficients with degree *n* and order *m*, λ is the longitude, θ is the geocentric latitude, and ${\bar{P}}_{nm}$ is the fully normalized associate Legendre function of the second kind.

### 2.2. Optical System Perspective of the Ellipsoid

For an optical system observing the planet at a position r, the projected surface image is a conic section [15], either a hyperbola or an ellipse. The general quadratic equation describes all conic sections:

(4) $$A\;x^{2}+B\;xy+C\;y^{2}+D\;x+F\;y+G=0,$$

where $B^{2}-4AC⋚0$ give an ellipse, a parabola, and a hyperbola, respectively.

The image plane depth would be typically represented by an optical system with focal length *f*, and so a vector similar to $p_{p}$ might be defined as $s={\left\{\begin{array}{ccc} x, & y, & f \end{array}\right\}}^{T}$. In matrix form,

(5) $$s^{T}\;C\;s=0,$$

where

(6) $$C=\frac{1}{2}\left[\begin{array}{ccc} 2A\;f^{2} & B\;f^{2} & D\;f\\ B\;f^{2} & 2C\;f^{2} & Fvf\\ D\;f & F\;f & 2G \end{array}\right].$$

Given that the planetary surface is being viewed by a camera in the body frame, it might be helpful to transform the ellipsoid treated in Section 2.1 to the camera frame using an orthogonal transformation matrix, $T_{pb}$, where the subscript implies a transformation from body frame “*b*” to planetary frame “*p*”. Equation (2) may then be rewritten as

(7) $$p_{b}^{T}T_{pb}^{\;T}A_{p}T_{pb}p_{b}=p_{b}^{T}A_{b}p_{b}=1,$$

where the transformation has been applied in the form $p_{p}=T_{pb}p_{b}$ and $A_{b}=T_{pb}^{\;T}A_{p}T_{pb}$.

The relation of the image plane position $s_{b}$ and planetary surface point $p_{b}$ is driven by the displacement of the body to the surface centre, $r_{b}$. This relation is simply

(8) $$p_{b}=ds_{b}+r_{b},$$

where $s_{b}$ is assumed to be of unit vector length and *d* is then the distance from the vehicle to the surface point. Substituting into Equation (7) produces

(9) $$1={\left(ds_{b}+r_{b}\right)}^{T}A\left(ds_{b}+r_{b}\right)=r_{b}^{T}A_{b}r_{b}+2dr_{b}^{T}A_{b}s_{b}+d^{2}s_{b}^{T}A_{b}s_{b}.$$

This expression takes the form of a quadratic equation which may be solved for *d*,

(10) $$d=-\frac{r_{b}^{T}A_{b}s_{b}}{s_{b}^{T}A_{b}s_{b}}.$$

Substituting back into Equation (9),

$$s_{b}^{T}\left(A_{b}r_{b}r_{b}^{T}A_{b}\right)s_{b}=s_{b}^{T}\left[\left(r_{b}^{T}A_{b}r_{b}-1\right)A_{b}\right]s_{b},$$

and rearranging,

(11) $$s_{b}^{T}M_{b}s_{b}=0,$$

where,

(12) $$M_{b}=A_{b}r_{b}r_{b}^{T}A_{b}-\left(r_{b}^{T}A_{b}r_{b}-1\right)A_{b}.$$

The matrix $M_{b}$ describes the perspective of the ellipsoid in the body frame of the optical sensor. This matrix contains some interesting properties that may be manipulated to identify the conic section shape visible to the sensor. It should be noted that this does not represent the projection on an image plane, which is dependent on the pointing direction.

### 2.3. Ellipsoid Shape and Horizon Dip

To further explore the shape observed in the image plane, considering if the sensor is not pointing directly at the ellipsoid, consider again Equation (11). Expanding out, this may be expressed as

(13) $$0=m_{11}x^{2}+2m_{12}xy+m_{22}y^{2}+2m_{13}fx+2m_{23}fy+m_{33}f^{2},$$

where $m_{ij}$ is the matrix term on row *i* and column *j*. This expression is the quadratic equation for Cartesian coordinates *x* and *y*. Depending on the coefficients, the expression may represent a circle, ellipse, parabola, or hyperbola. The hyperbolic expression is reflective of cases when the sensor is close to the surface, as is the case for a maritime vessel.

The approximate “dip” in the horizon caused by the Earth’s surface curvature is dependent on the distance to the surface and the view field size. Considering the Earth’s surface is captured using a camera sensor, the horizon dip may be expressed in camera pixels, and so another dependent is the sensor resolution.

**Figure 2:**
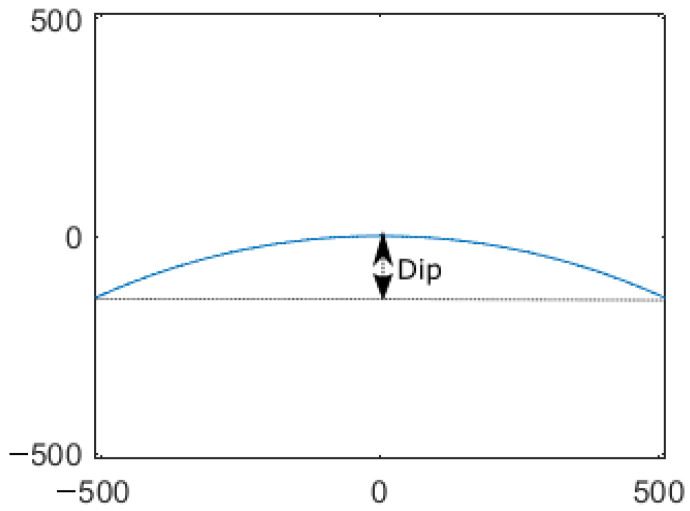
Horizon dip of the body shape function. Axes are in pixels.

To calculate this accurately, a chord line to the curve is drawn across the image boundaries. The distance from which the line and curve are greatest is the horizon dip. This fit is illustrated for an image of resolution 1024×1024 px in Figure 2.

**Figure 3:**
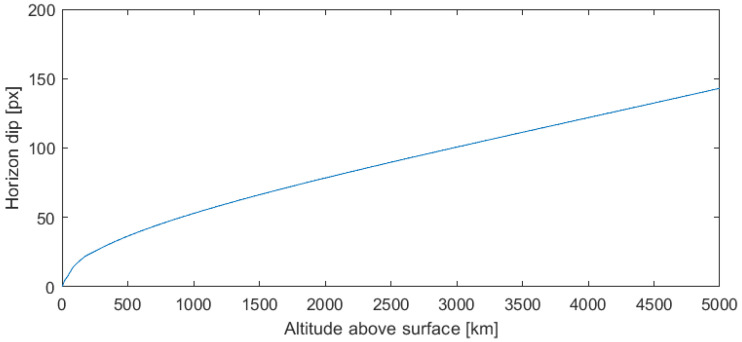
Horizon dip as the craft descends in altitude towards Earth.

Simulating for various altitudes and calculating the maximum difference between the chord to the curve, the horizon dip is presented in Figure 3. This highlights that for low altitudes, the dip in the horizon is indistinguishable from the sensor noise, which might be a few pixels in magnitude for each sensed horizon position. This noise may also be indistinguishable from slight changes to the horizon’s surface with the geoid.

To justify the negligible noise caused by the geoid’s variation to the ellipsoid, consider the angular difference in the horizon from the zenith to the horizon pointing vector, i.e., between r and s. Using Pythagoras, considering a triangle constructed by a line pointing from the Earth’s origin to the ship and another line pointing to the perpendicular of s, the angle might be expressed by

(14) $$\sin\alpha =\frac{R_{M}^{2}}{{\left(R_{M}+\delta \right)}^{2}}.$$

where $R_{M}$ is the meridional radius for the ellipsoid, defined by

(15) $$R_{M}=\frac{a(1-e^{2})}{{\left(1-e^{2}\sin^{2}\phi \right)}^{3/2}}.$$

If there is a variation in the meridional radius caused by the geoid, then using linear error propagation, it may be expressed as an equivalent error in the angle by

(16) $$\sigma_{\alpha }=\frac{\partial \alpha }{\partial R_{M}}\sigma_{R_{M}}=\frac{2R_{M}\delta }{\left(R_{M}+\delta \right)\sqrt{{\left(R_{M}+\delta \right)}^{4}-R_{M}^{4}}}\sigma_{R_{M}}.$$

Considering the geoid variation range does not exceed ±100 m, this is equivalent to the standard deviation of angle change in α by 0.0041 arc-secs and thus would be negligible for most standard camera optics.

### 2.4. Measuring Stars on the Earth’s Surface

Measuring stars on Earth has been a common problem for the navigator since ancient history when early civilizations such as the Polynesians used the night sky to navigate across land and sea. The celestial map has since been treated as a tool for both determining position and attitude. In this work, the orientation determination properties of stars and their constellations will be treated and adopted. Known as star tracking, in the form of a star tracker sensor, the orientation matrix measured is utilized alongside the horizon to determine a position and is the topic of Section 3.

Star tracker orientation determination for space applications has been well studied for the last 60 years since Apollo, and many models exist to estimate sensor pointing from a sequence of star measurements [19,20,21,22,23]. State-of-the-art systems can deliver attitude accuracies at 1 arc-sec, especially when combined with a gyroscope [20]. However, this might be more difficult on Earth-bound ships.

The key challenge for the star tracker on Earth is the effects if the atmosphere, both in terms of optical effects caused by the varying compositional layers of the atmosphere and visibility due to cloud cover and ambient light. The atmospheric deflection makes the star appear closer to the zenith. This effect increases towards the horizon, with a maximum shift close to 34 arc-min at this extremity [24]. The other effect is astronomical seeing, where varying winds and air currents across different atmospheric layers distort the known refraction. However, this noise is only approximately 1 arc-sec [25].

The effect of atmospheric deflection has been a problem treated and solved in the 19th century by Gauss, using three measured stars, with known right ascension and declination in the celestial sphere, and time [26]. Since star identification has been solved by pattern recognition alone [19], the defection effect may then be corrected with absolute precision under a standard atmosphere. The considered noise of deflection may then be 1 arc-sec by astronomical seeing. In addition, atmospheric deflection is a known function of the star elevation over the local horizon, and the local horizon can always be computed by two orthogonal inclinometers, as suggested in Ref. [14].

Sky visibility is more challenging and highly unpredictable. Optical filters have been installed and tested on cameras to permit better daytime operations, targeting the shortwave infrared [27,28]. Stars were measured on a balloon-borne platform, delivering below sub-arc-second performances. Cloud cover leads to nearly no operation of the star tracker, and poor visibility cause by low-lying clouds can also disrupt the horizon. It is considered that the proposed techniques in this work cannot operate in those conditions. However, the suggested technique of using two inclinometers in association may help [14].

On the ground, star trackers have been both evaluated and tested on Earth in previous work of the authors [29,30,31]. Both have demonstrated arc-second precision. The estimated accuracy of the star tracker is simulated at approximately σ=10 arc-sec, as has been demonstrated is obtainable from previous testing. This figure is a conservative estimate and considers the lower-end spectrum of sensors that employ commercial-off-the-shelf components [23].

## 3. Least-Squares Formulations

The surface descriptions treated previously may be manipulated to provide an estimate of the sensor position based on a series of measured horizon points. Four models are introduced. The first is a simple Non-Linear Least Squares (NLLS) approach to the ellipsoid description introduced through Equation (11) proposed in [16] for a three-dimensional position, the second is based on the Christian–Robinson method [16], and the last two take advantage of a usually known surface height and near linear horizon surface.

In each model, the transformation matrix $T_{pb}$ must also be estimated. This is derived by star measurements, which provide knowledge of the difference between the inertial celestial frame and the sensor body frame, creating a transformation matrix $T_{ib}$. Using time knowledge and well-known models of the Earth’s rotation and nutation, the frame transformation from inertial to fixed planetary $T_{pi}$ is also known. Thus, the required transformation matrix is calculated by $T_{pb}=T_{pb}\left(q\right)=T_{pi}\;T_{ib}$, where q is the quaternion description.

### 3.1. Position by Nonlinear Least-Squares

The nonlinear least-squares solution is obtained using the treatment of Equation (11) maintained in the implicit state. To avoid formulations of higher-order tensors, this can be treated in the planetary frame. Rewriting in this frame, we obtain

(17) $${\left(T_{pb}\left(q\right)\;s\right)}^{T}\;M_{p}\;T_{pb}\left(q\right)\;s=0,$$

where

$$M_{p}=A_{p}r_{p}r_{p}^{T}A_{p}-\left(r_{p}^{T}A_{p}r_{p}-1\right)A_{p}.$$

For the rest of this article, all parameters will be considered in the planetary frame unless explicitly stated, where horizon measurements, s, have been transformed by the estimated attitude by the star tracker.

First, considering the state vector derivatives, it might be helpful to write out Equation (11) as

$$s^{T}Arr^{T}As-s^{T}\left(r^{T}Ar-1\right)As=0.$$

For the derivative of the position vector, we may then consider each term. The first term is treated as

$$\begin{array}{c} s^{T}Arr^{T}As={\left(A^{T}s\right)}^{T}rr^{T}As=w^{T}rr^{T}w \end{array}$$

where $w=As=A^{T}s={\left\{\begin{array}{ccc} w_{1}, & w_{2}, & w_{3} \end{array}\right\}}^{T}$. Taking the derivative in terms of r obtains

$$\begin{array}{c} \frac{\partial }{\partial r}\left(s^{T}Arr^{T}As\right)=2r^{T}ww^{T}=2r^{T}As{\left(As\right)}^{T}. \end{array}$$

Similarly, expanding the second term,

$$\begin{array}{c} s^{T}\left(r^{T}Ar-1\right)As=s^{T}\left(r^{T}Ar\right)As-s^{T}As=\left(r^{T}Ar\right)s^{T}As-s^{T}As, \end{array}$$

and taking the derivative in terms of r,

$$\begin{array}{cc} \frac{\partial }{\partial r}\left[\left(r^{T}Ar\right)s^{T}As-s^{T}As\right] & =2rAs^{T}As, \end{array}$$

using again the knowledge that $A=A^{T}$. Combining the two terms produces

(18) $$\frac{\partial }{\partial r}\left(s^{T}Ms\right)=2r^{T}As{\left(As\right)}^{T}-2rAs^{T}As.$$

The nonlinear least-squares technique is an iterative solution, which, for the implicit model function $h=s^{T}Ms=0$, is given by

(19) $$x_{k+1}=x_{k}-{\left(H^{\;T}H\right)}^{-1}H^{\;T}h,$$

where the state estimate is x=r and *k* is the iteration number. This technique is dependent on a good initial guess of the solution to achieve a correct convergence, given this solution is not optimal by its highly nonlinear nature.

### 3.2. Christian-Robinson Method

The Christian–Robinson technique is considered one of the most successful techniques for optical navigation in spacecraft, providing a much stronger performance compared to traditional ellipse-fitting methodologies [15]. The method returns to Equation (11), rewritten here for convenience:

(20) $$s_{i}^{T}Ms_{i}=s_{i}^{T}Arr^{T}As_{i}-s_{i}^{T}\left(r^{T}Ar-1\right)As_{i}=0.$$

The solution harnesses the transformation of this expression into Cholesky factorization space, which is given by

(21) $$\bar{r}=Ur\;\mathrm{and}\;{\bar{s}}_{i}=Us_{i},$$

where

(22) $$A=U^{T}U.$$

Thus, we can rewrite the factorization as

(23) $${\bar{s}}_{i}^{T}I_{3\times 3}\bar{r}{\bar{r}}^{T}I_{3\times 3}{\bar{s}}_{i}-{\bar{s}}_{i}^{T}\left({\bar{r}}^{T}I_{3\times 3}\bar{r}-1\right)I_{3\times 3}{\bar{s}}_{i}={\bar{s}}_{i}^{T}\bar{r}{\bar{r}}^{T}{\bar{s}}_{i}-{\bar{s}}_{i}^{T}\left({\bar{r}}^{T}\bar{r}-1\right){\bar{s}}_{i}=0,$$

where the variables of $\bar{r}$ and ${\bar{s}}_{i}$ are now in Cholesky space. The shape matrix in this space has been replaced with an identity matrix $I_{3\times 3}$, and so the observed body is no longer an ellipsoid, but a much simpler unit-sphere.

Given the transformed geometry, the angle between the horizon line-of-sight direction vectors ${\bar{s}}_{i}$ and the relative position vector $\bar{r}$ are the same, and so,

(24) $${\bar{s}}_{i}^{T}\bar{r}=-\bar{r}\cos\bar{\theta },$$

where it is assumed ${\bar{s}}_{i}$ has been normalized to a unit vector and $\bar{r}$ is the magnitude of the transformed position vector, i.e., $\bar{r}=\left|\right|\bar{r}\left|\right|$. Define a new quantity

(25) $$n=\frac{-1}{\bar{r}\cos\bar{\theta }}\bar{r},$$

which permits the expression to be rewritten as

(26) $${\bar{s}}_{i}^{T}n=1.$$

The new quantity n is essentially a pointing vector to the unit-sphere from the transformed observer position.

The problem has now been reduced to a simple linear least-squares problem. Given a set of *m* horizon points, the problem may be expressed using

(27) $$Hn=1_{m\times 1},$$

where

(28) $$H=\left[\begin{array}{c} {\bar{s}}_{1}^{T}\\ \vdots \\ {\bar{s}}_{m}^{T} \end{array}\right].$$

Reference [15] recommends that this problem be solved by total least-squares, which involves the Singular Value Decomposition (SVD) of $\left[\begin{array}{c} H1_{m\times 1} \end{array}\right]$, and n is calculated by the eigenvector corresponding to the maximum eigenvalue, with $n=\frac{V_{1:3,4}}{V_{4,4}}$.

Solving for n and working backward allows for a solution to r, the position of the sensor, and the desired state output. This may be simply calculated from

(29) $$r=-{\left(n^{T}n-1\right)}^{-1/2}U^{-1}n.$$

### 3.3. Near Linear Horizon

Given the analysis presented in Section 2.3, when a sensor is near the surface, there is a very small dip from the horizon over the image field of view. This function may nearly be treated as a straight line.

**Figure 4:**
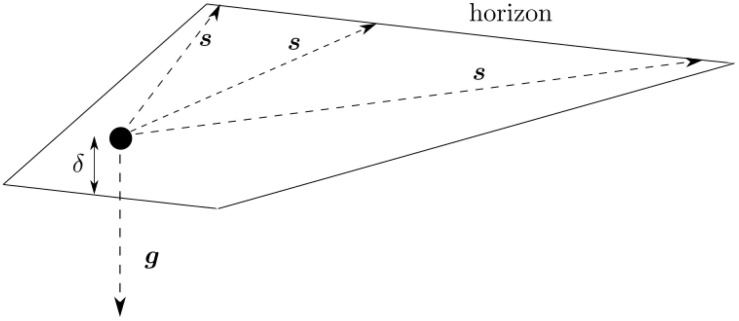
Illustration of a nearly flat horizon, where the object is slightly elevated above the surface at a height δ.

The vectors pointing towards the horizon, s, may be approximated as very nearly perpendicular to the gravitational field vector, g. This model is illustrated in Figure 4. The object is slightly elevated above the surface at a height δ.

Using this approximation, alongside the approximate gravity vector in a geocentric model, where

(30) $$g={\left\{\begin{array}{ccc} \cos\lambda \cos\phi , & \sin\lambda \cos\phi , & \sin\phi \end{array}\right\}}^{T},$$

the position of the user in terms of latitude and longitude may be known from only the horizon measurements and a known pointing attitude. Recalling that first the body measurements of the horizon need to be transformed to the planetary frame, the final model expression is

(31) $$h\left(\lambda ,\phi ,s\right)=s^{T}\left\{\begin{array}{c} \cos\lambda \cos\phi \\ \sin\lambda \cos\phi \\ \sin\phi \end{array}\right\}=0.$$

This model is in an implicit form, and so may be solved by the implicit form of a nonlinear least-squares, first described in Section 3.1.

The Jacobian is formulated by

(32) $$H={\left[\begin{array}{cc} \frac{\partial h}{\partial \lambda }, & \frac{\partial h}{\partial \phi } \end{array}\right]}_{i},$$

where

$$\begin{array}{cc} \frac{\partial h}{\partial \lambda } & ={\left(T_{pb}s\right)}^{T}\left\{\begin{array}{c} -\sin\lambda \cos\phi \\ \cos\lambda \cos\phi \\ 0 \end{array}\right\}\\ \frac{\partial h}{\partial \phi } & ={\left(T_{pb}s\right)}^{T}\left\{\begin{array}{c} -\cos\lambda \sin\phi \\ -\sin\lambda \sin\phi \\ \cos\phi \end{array}\right\}. \end{array}$$

Note that this function has made the assumption that the two vectors are perpendicular. As δ and/or field of view increases, s becomes much less perpendicular but at a small angle θ. This angle was also encountered in Section 3.2 through the transform Cholesky factorization space. The angle is dependent on the ellipsoid being observed. For this simplistic case in the geocentric frame, the angle is not dependent on the position, as all radii are the same. The correction is derived from Equation (14) for a standard Earth radius *R*,

(33) $$h=\sqrt{\frac{\delta (\delta +2R)}{\delta +R}},$$

which is applied to Equation (31).

### 3.4. Geodetic Horizon

Section 3.3 does not account for ellipsoidal shape in the direction of latitude. This is not representative and so should not be discarded lightly, even though the dip is barely noticeable as considered in Section 2.3.

To account for the curved horizon shape close to the surface, the ellipsoidal model of the Earth should be adopted, as in previous models described in Section 3.1. However, the advantages of using a near-linear horizon should also be considered.

Recalling that the description of an ellipsoidal point on the Earth’s surface p is given by $p^{T}Ap=1$, then for a sensor on the surface, $p=\left||p|\right|\hat{r}=\rho \hat{r}$, where $\hat{r}$ is a unit vector in the direction of the sensor position, it may be written as

(34) $$p^{T}Ap=\rho^{2}{\hat{r}}^{T}A\hat{r}=1.$$

Consider now that the sensor is elevated from the surface by a height δ, so $r=\left(\rho +\delta \right)\hat{r}$. The ellipsoidal body matrix is then written as

$$\begin{array}{cc} M & ={\left(\rho +\delta \right)}^{2}A\hat{r}{\hat{r}}^{T}A-\left[{\left(\rho +\delta \right)}^{2}{\hat{r}}^{T}A\hat{r}-1\right]A\\ \; & ={\left(\frac{1}{{\left({\hat{r}}^{T}A\hat{r}\right)}^{1/2}}+\delta \right)}^{2}A\hat{r}{\hat{r}}^{T}A-\left[{\left(\frac{1}{{\left({\hat{r}}^{T}A\hat{r}\right)}^{1/2}}+\delta \right)}^{2}{\hat{r}}^{T}A\hat{r}-1\right]A\\ \; & =\chi \left(\hat{r}\right)\tilde{M}\left(\hat{r}\right)+A, \end{array}$$

where

$$\begin{array}{cc} \chi (\hat{r}) & =\frac{1}{{\hat{r}}^{T}A\hat{r}}+\frac{2\delta }{{\left({\hat{r}}^{T}A\hat{r}\right)}^{1/2}}+\delta^{2}\\ \tilde{M}\left(\hat{r}\right) & =A\hat{r}{\hat{r}}^{T}A-{\hat{r}}^{T}A\hat{r}A. \end{array}$$

As in the near-linear horizon model of Section 3.3, the position vector may be described using the geocentric longitude and latitude, as was considered for the gravity vector:

(35) $$r=r\hat{r}=\left(\rho +\delta \right){\left\{\begin{array}{ccc} \cos\lambda \cos\phi , & \sin\lambda \cos\phi , & \sin\phi \end{array}\right\}}^{T}.$$

The latitude and longitude will be the desired state vectors, and the problem may be formulated with an NLLS approach. The model may be written as

(36) $$h\left(\lambda ,\phi ,s\right)=s^{T}Ms=\chi \left(\hat{r}\right)s^{T}\tilde{M}\left(\hat{r}\right)s+s^{T}As=0.$$

To determine the Jacobian, the chain rule may be adopted for the state vector, letting the row for the *k*-th become

(37) $$H={\left[\begin{array}{cc} \frac{\partial h}{\partial \lambda }, & \frac{\partial h}{\partial \phi } \end{array}\right]}_{k}={\left[\begin{array}{cc} \frac{\partial h}{\partial \hat{r}}\cdot \frac{\partial \hat{r}}{\partial \lambda }, & \frac{\partial h}{\partial \hat{r}}\cdot \frac{\partial \hat{r}}{\partial \phi } \end{array}\right]}_{k}.$$

The solution for the implicit NLLS is calculated by Equation (19).

Evaluating for $\frac{\partial h}{\partial \hat{r}}$ leads to use of the product rule

(38) $$\frac{\partial h}{\partial \hat{r}}=\frac{\partial \chi }{\partial \hat{r}}s^{T}\tilde{M}\left(\hat{r}\right)s+\chi \left(\hat{r}\right)\frac{\partial }{\partial \hat{r}}\left(s^{T}\tilde{M}\left(\hat{r}\right)s\right).$$

The derivative of $s^{T}\tilde{M}\left(\hat{r}\right)s$ was calculated in Section 3.1, but the r is replaced by $\hat{r}$. The derivative of $\chi (\hat{r})$ is expressed as

(39) $$\frac{\partial \chi }{\partial \hat{r}}=\frac{-2}{{\left({\hat{r}}^{T}A\hat{r}\right)}^{2}}{\hat{r}}^{T}A-\frac{2\delta }{{\left({\hat{r}}^{T}A\hat{r}\right)}^{3/2}}{\hat{r}}^{T}A.$$

The derivative of $\hat{r}$ has already been calculated in the form of Equation (32), where the leading terms to the vector may be removed.

## 4. Simulation Methodology and Performance

To demonstrate the performance of each approach and the importance of using the known sensor altitude and consideration of the geodetic horizon, a series of Monte Carlo simulations are performed for a theoretic vessel at various positions on the Earth’s surface. This section describes the model parameters, noise treatment, and estimated performance of the techniques described.

### 4.1. Model Parameters

A theoretical optical-based sensor is considered. The parameters of the sensor are presented in Table 1. The sensor is placed on a ship with the horizon pointing directly toward the line.

**Figure 5:**
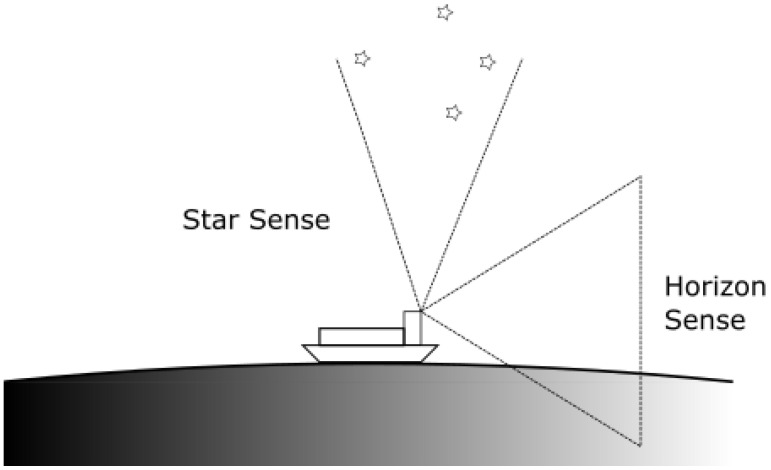
Orientation of the horizon and star sense optical assembly.

Placed orthogonal to the optical sensor at the zenith is a star tracker. The star tracker is introduced in Section 2.4 and is assumed to have a theoretical performance of σ=10 arc-sec. The orientation is illustrated in Figure 5.

**Figure 6:**
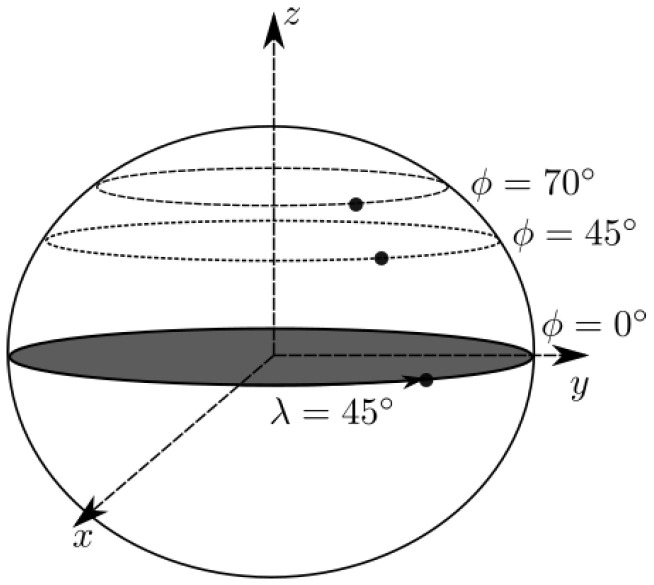
Illustration of the varying latitude scenarios treated in this paper.

The model is simulated for several scenarios of a sensor placed on the Earth’s ellipsoid. Each treats a different shape of the horizon curve, placed at different latitudes. The solution should have no dependence on the longitude. The position of the vessel on the ellipsoid is illustrated in Figure 6.

### 4.2. Noise Model

A measured position of the horizon is represented by the sub-pixel coordinates of the point (α,γ). The sensor frame unit vector is related to the sub-pixel coordinates by

(40) $$\alpha =-f\frac{s_{x}}{s_{z}},\;\gamma =-f\frac{s_{y}}{s_{z}},$$

where *f* is the focal length. The focal length is related to the sensor resolution β and field of view FOV by the relation

(41) $$f=\frac{\beta }{2\tan(FOV/2)}.$$

The reverse relation uses the unit vector normalization condition |s|=1. So, the s unit vector may be calculated by

(42) $$s=\frac{1}{\sqrt{\alpha^{2}+\gamma^{2}+f^{2}}}\left\{\begin{array}{c} -\alpha \\ -\gamma \\ f \end{array}\right\}.$$

A common covariance model for star trackers is adopted [32,33] using the measured image coordinates (α,γ). The horizon points may be treated similarly to star points, approximating the distribution to a Gaussian. The covariance is expressed by

(43) $$R=\frac{\sigma^{2}}{1+\alpha^{2}+\beta^{2}}\left[\begin{array}{cc} {\left(1+\alpha^{2}\right)}^{2} & {\left(\alpha \gamma \right)}^{2}\\ {\left(\alpha \gamma \right)}^{2} & {\left(1+\gamma^{2}\right)}^{2} \end{array}\right].$$

This can then be introduced to each measured horizon position. It is assumed that there are no camera distortion effects.

### 4.3. Model Performance

**Figure 7:**
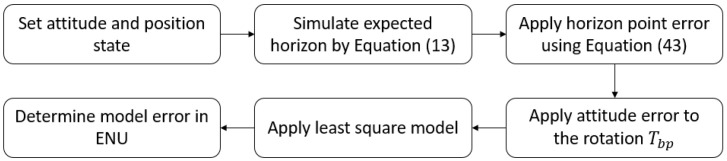
Software implementation methodology to estimate model performance.

The four models described in Section 3 are compared and contrasted. The implementation will follow the sequence illustrated in Figure 7.

As introduced, three different latitudes are considered. The results are presented initially for the latitude of 0${}^{\circ }$ and 45${}^{\circ }$, which present the best and worst possible horizon distribution shapes for the sensor. The results are then treated for a latitude of 70${}^{\circ }$, also including a complete probability distribution.

**Figure 8:**
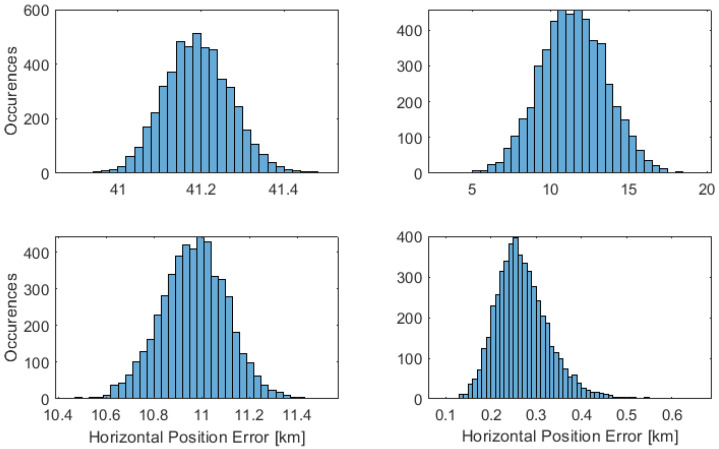
Horizontal performance of a vessel at 70${}^{\circ }$ latitude. The placement of each technique in the figure is labelled as set out in Table 2.

**Figure 9:**
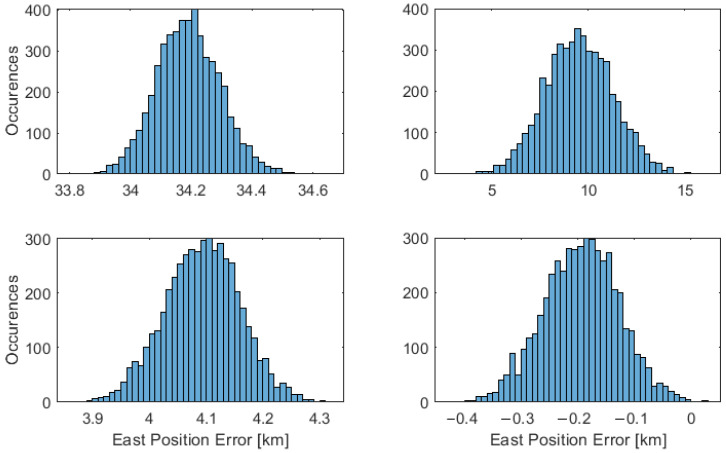
East performance of a vessel at 70${}^{\circ }$ latitude. The placement of each technique in the figure is labelled as set out in Table 2.

**Figure 10:**
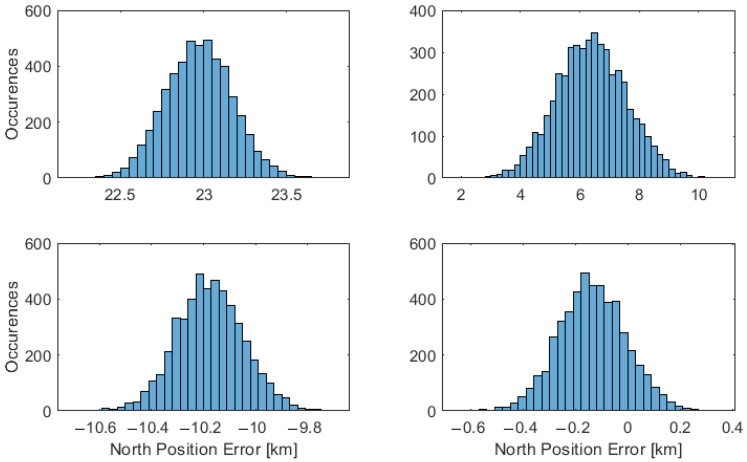
North performance of a vessel at 70${}^{\circ }$ latitude. The placement of each technique in the figure is labelled as set out in Table 2.

The probability distribution is presented considering the horizontal, east, and north directions, with an error difference established from the position truth. The results are illustrated in Figure 8, Figure 9 and Figure 10, with the placement of each figure highlighted in Table 2. The performance statistics are presented in Table 3, with the mean and Standard Deviation (STD) presented.

The presented results highlight two key features. The first is the benefit that knowledge of the sensor height holds to a horizon fit solution, where the desired state only consists of latitude and longitude. Sensor height knowledge provides implied information about the depth of the observable horizon, aiding the solution. Techniques 1 and 2 each attempt to solve for three unknowns.

The second is the assumption of ellipsoidal curvature in the solution. Techniques that treat the horizon as a hyperbola or ellipse without prior knowledge of height cannot accurately determine curvature, as only a small arc is observed of the conic. Technique 3, even though the sensor height is known, does not include realistic information on the observed horizon curvature. It is thus not suitable for implementation.

This leads to be the best solution being the technique of geodetic horizon, Technique 4, introduced in Section 3.4. The factor improvement in performance is about 100 m compared to the other three techniques.

There is a bias present in the solution caused by the limitations and assumptions of each model. This bias is based on the observed geometry, dependent on the current location and its local environment, so it cannot be predicted and corrected.

The performance derived demonstrates a marked improvement from the traditional sextant, with the method of geodetic horizon achieving a 1-sigma precision in the hectometre scale. It was also justified that geoid deviation, otherwise described as gravitational anomalies, holds little impact on this solution.

## 5. Conclusions

In this work, performances are demonstrated that exceed the traditional approaches of a sextant, delivering an accuracy and precision under 100 m in the horizontal plane. However, Global Navigation Satellite System (GNSS) remains the most accurate form of navigation reference for a ship, and so this technique may act as an alternative or back-up solution or as a sanity-check method to validate the GPS’s estimation.

The best approach considers a geodetic-based horizon. Solutions less than this geometric approximation deliver a much poorer performance. A bias remains in the solution, caused by unknowns of the local horizon’s geodetic curvature.

Additional knowledge of the oceanic environment should also be accounted for, such as waves and currents. Isolation of the wave modal frequency may assist in ensuring minimal errors derived by these external influences. This may be accomplished by introducing inclinometers as part of the sensor suite.

It should also be considered that the proposed technique may not operate under cloud cover, and so alternative techniques would be required to navigate and, in absence of reliable GPS signals, continue to navigate in open-loop as a short-term solution. Integration of a pair of inclinometers may assist in such conditions. Dead-reckoning may also be applied in such environments, combined with a filter-like technique, such as a Kalman filter. The sensor suite may then be introduced to real-time operations for many marine environments.

The authors also seek to further verify these algorithms employing real ocean and star images off the coast. The Sydney coastline has been chosen for this and will be a topic of further work. The ambition is to develop and test an integrated sensor suite of the star tracker, horizon sensor, and two inclinometers for deployment on a ship.

## Figures and Tables

**Table 1:** Horizon optical sensor parameters.

Parameter	Value
Field of view	40${}^{\circ }$
Sensor resolution	2048 × 2048 px

**Table 2:** Position technique labels.

Number	Technique	Section	Placement
1	Position by nonlinear least-squares	Section 3.1	Top-Left
2	Christian–Robinson Method	Section 3.2	Top-Right
3	Near Linear Horizon	Section 3.3	Bottom-Left
4	Geodetic Horizon	Section 3.4	Bottom-Right

**Table 3:** Position performance at 0${}^{\circ }$, 45${}^{\circ }$, and 70${}^{\circ }$ latitude, from top to bottom, rounded to the nearest 10 m accuracy. Each result is presented with the mean followed by the standard deviation in brackets.

Technique Number	Latitude [deg]	Horizontal [km]	East [km]	North [km]
1	0	35.78 (0.02)	35.78 (0.02)	−0.01 (0.37)
2	0	9.00 (6.79)	0.17 (11.24)	−0.04 (0.86)
3	0	0.22 (0.15)	−0.05 (0.15)	0.07 (0.25)
4	0	0.20 (0.15)	0.00 (0.02)	−0.01 (0.25)
1	45	30.49 (0.07)	13.51 (0.035)	−27.34 (0.24)
2	45	38.24 (0.04)	20.69 (0.31)	−32.16 (0.16)
3	45	21.30 (0.11)	−0.08 (0.20)	21.29 (0.11)
4	45	0.24 (0.12)	−0.00 (0.20)	0.14 (0.11)
1	70	41.19 (0.08)	34.19 (0.11)	22.97 (0.20)
2	70	11.47 (2.11)	9.52 1.75)	6.39 (1.18)
3	70	10.97 (0.14)	4.09 (0.07)	−10.18 (0.13)
4	70	0.27 (0.06)	−0.19 (0.07)	−0.13 (0.13)
